# [Retracted] Tanshinone IIA enhances the inhibitory effect of imatinib on proliferation and motility of acute leukemia cell line TIB‑152 *in vivo* and *in vitro* by inhibiting the PI3K/AKT/mTOR signaling pathway

**DOI:** 10.3892/or.2024.8725

**Published:** 2024-03-11

**Authors:** Zhi Teng, Shijuan Xu, Qin Lei

Oncol Rep 43: 503–515, 2020; DOI: 10.3892/or.2019.7453

Following the publication of the above article, a concerned reader drew to the Editor's attention that the Transwell cell invasion assay data featured in Figs. 2D and 4E, certain of the tumor images in Fig. 5A and the TUNEL assay data in Fig. 5C were strikingly similar to data appearing in different form in other articles written by different authors at different research institutes that had either already been published elsewhere prior to the submission of this paper to *Oncology Reports*, or which under consideration for publication at around the same time (some papers of which have been retracted).

In view of the fact that certain of these data had already apparently been published prior to the submission of this article for publication, the Editor of *Oncology Reports* has decided that this paper should be retracted from the Journal. The authors were asked for an explanation to account for these concerns, but the Editorial Office did not receive a reply. The Editor apologizes to the readership for any inconvenience caused.

